# 

**Published:** 1874-04

**Authors:** 


### Contents of April Number.

### ORIGINAL DEPARTMENT.

ART. I—The Treatment of Uterine Flexions. By Ely Van De Warker, M. D. .... 321

ART. II—Observations in Electro-Therapeutics. By A. D. Rockwell, M. D. .... 333

ART. III—Case of Fracture of both bones of the left Leg, Treated in Wood's Hammock Splint. By G. W. Bowen, M. D. .... 342

### MISCELLANEOUS.

Transfusion, .... 344

Reports on Life Assurance, .... 348

Mercury in Syphilis, .... 350

Successful Abdominal Section for Intussusception, .... 351

### EDITORIAL DEPARTMENT.

Meeting of the American Medical Association, .... 353

Mortality Statistics of Buffalo, .... 354

Guarana—Election of Officers—Chicago Journal of Nervous Diseases, .... 355

Books Reviewed:

Dunglison's Medical Dictionary .... 356

Griffith's Formulary .... 356

Gunshot Wounds of the Abdomen. By Eugene Peugnet, M. D. .... 357

Bellamy's Guide to Surgical Anatomy, .... 358

The Sphygmograph. By Edgar Holden, M. D. .... 358

Annual Report of the Supervising Surgeon of the U. S. Marine Hospital Service .... 359

Books and Pamphlets Received .... 359

